# Breast cancer mortality as a function of age

**DOI:** 10.18632/aging.203881

**Published:** 2022-02-08

**Authors:** Azadeh Nasrazadani, Juan Luis Gomez Marti, Kevin E. Kip, Oscar C. Marroquin, Lara Lemon, Steve D. Shapiro, Adam M. Brufsky

**Affiliations:** 1 Division of Medical Oncology, Department of Medicine, University of Pittsburgh School of Medicine, Pittsburgh, PA 15213, USA; 2 Department of Pathology, University of Pittsburgh School of Medicine, Pittsburgh, PA 15213, USA; 3 Clinical Analytics, University of Pittsburgh Medical Center, Pittsburgh, PA 15213, USA; 4 Department of Obstetrics, Gynecology, and Reproductive Sciences, University of Pittsburgh Medical Center, Pittsburgh, PA 15213, USA; 5 Keck Medicine of USC, University of Southern California, Los Angeles, CA 90007, USA

**Keywords:** breast cancer, aging, mortality, expected survival, disparities, elderly

## Abstract

Background: Incidence of breast cancer (BC) in US women continues to increase with age as the strongest risk factor. We aimed to compare clinical, pathological and sociological variables associated to BC diagnosis, as well as the relative mortality rates of BC patients compared to the general US population.

Methods: We performed a retrospective, single-institution study evaluating 52,509 patients diagnosed with unilateral BC at the University of Pittsburgh Medical Center (UPMC) between 1990–2020. Primary outcome was death from any cause with cancer recurrence as a secondary outcome, evaluated for 4 age groups: 20–44, 45–55, 56–69, and 70–90. A dataset of expected mortality for women in the general population over a 10-year period was constructed using the Surveillance, Epidemiology, and End Results (SEER) Program. Observed vs. expected mortality and standardized mortality ratios (SMR) for each age group were calculated.

Results: Youngest patients with BC demonstrated the highest SMR at 10-year follow-up from time of diagnosis compared to the general US population (SMR 9.68, 95% CI: 8.99to 10.42), and remained highest compared to other age groups when analysis was limited to Stage 0/1 disease (10-year SMR 3.11, 95% CI: 2.54 to 3.76). SMRs decreased with increasing age at diagnosis with an SMR <1.0 in patients diagnosed with stage 0/1 at ages 70–90 at 5-year follow-up.

Conclusions: Younger BC patients have the highest SMR which declines gradually with age. In the elderly, lower stage 0/1 SMR’s are found compared to the general population, suggesting the possibility of an associated protective effect.

## INTRODUCTION

As life expectancy in the US has steadily increased up until prior to the COVID-19 pandemic [1], trends in incidence of BC, particularly the estrogen receptor (ER) positive subtypes, are similarly following as a consequence of aging [2]. While BC remains the most highly incident cancer diagnosed in US women, the female BC death rate has declined significantly over the preceding years [3]. The overall survival of elderly women with BC is predictably worse to that of younger counterparts given higher rates of comorbidities and frailty due to older age. However, when evaluating BC specific survival (BCSS), elderly patients have significantly better outcomes yet with the youngest patients demonstrating the worst BCSS rates [4].

Lower BC-associated mortality in the elderly is notable considering challenges faced with providing aggressive standard of care therapies when limited by performance status and frailty measures, suggesting the development of relatively more indolent disease in the elderly with a less aggressive course. Congruently, multiple studies have characterized BCs in younger patients with higher histological grade, larger size, low or absent expression of ER and/or progesterone receptors (PR), and greater lymph node (LN) positivity [5–10]; while LN positivity in patients with early stage disease is found to be decreased with increasing age [11].

To gain insight into the relative lethality of BC in women diagnosed at younger versus older ages, we sought to compare mortality rates among BC patients as compared to mortality rates in the general population, matched by age and race (i.e., observed vs. expected mortality). With the presumption that BCs diagnosed at younger ages represent a more severe clinical entity from disease diagnosed at older ages, a larger differential in observed to expected mortality was postulated in younger women diagnosed with BC. In contrast, in elderly patients with BC, we hypothesized little to no difference in observed to expected mortality due to a potentially less aggressive disease course, coupled with overall high competing (non-BC) causes of mortality in older women with and without BC.

## METHODS

### Sources of data

Data in the UPMC electronic medical record (EMR) systems encompasses detailed sociodemographic, diagnostic, surgical and other related procedures, prescriptions, and billing data; on all outpatient and in-hospital encounters. Diagnoses and procedures are coded based on the International Classification of Diseases, Ninth and Tenth revisions (ICD-9 and ICD-10, respectively). As previously described [12], we linked the primary data sources using common variables (deidentified) within the UPMC data ecosystem aggregated in its Clinical Data Warehouse (CDW) that include: (i) Medipac, the admit, discharge and transfer registration and hospital-based billing system; (ii) Cerner, the inpatient EMR for relevant clinical information for bedded patients at a UPMC inpatient hospital; (iii) Epic, the UPMC EMR for ambulatory office visits owned by UPMC; and (iv) Aria, the EMR utilized in most ambulatory Cancer Centers at UPMC for both radiation oncology and medical oncology. These data were augmented with UPMC cancer registry data, acquired using Elekta METRIQ registry software.

### Patient population

We studied 52,509 female patients from 60 hospital/outpatient facilities within the UPMC system with an initial diagnosis of unilateral BC between 20 to 90 years of age spanning the period of January 2, 1990 to July 15, 2020. The last date of contact/documentation within the EMR system was September 4, 2020, with a median of 5.9 years of follow-up to last contact or death (interquartile range: 2.3 to 11.0 years). Our study received formal ethics approval by the UPMC Ethics and Quality Improvement Review Committee (Project ID 2882), the ethics/oversight body for ensuring patient confidentiality and consent (including waiver of consent) and analysis and dissemination of deidentified data within the UPMC system.

### Outcomes

The primary outcome was death from any cause, and recurrence (local or distant) of BC was tabulated as a secondary outcome. Observed to expected mortality was reported as SMR. We assessed in-hospital observed mortality by the discharge disposition of “Ceased to Breathe” sourced from the inpatient Medical Record System. These deaths were augmented with those externally identified with the Death Master File (DMF) from the Social Security Administration (SSA) (NTIS 2020) as an external data source. The investigators were blinded to mortality ascertainment within the UPMC system. Expected mortality was determined as described in statistical methods below.

### Explanatory variables

For comparison of mortality by age at BC diagnosis, we categorized the study cohort into 4 groups as follows: 20 to 44 years (i.e., pre-menopausal); 45 to 55 years (i.e., peri-menopausal); 56 to 69 years (i.e., post-menopausal); 70 to 90 years (i.e., late post-menopausal). For comparisons by age groups at diagnosis, we considered socio-demographic variables, selected medical comorbidities, anthropometric/laboratory values, and medication use, along with specific cancer characteristics including stage, tumor grade, histology and severity, hormone receptor status, and cancer treatment approaches employed. The systemic immune-inflammatory index (SII) was calculated by the platelet count × neutrophil/lymphocyte ratio (NLR) [13]. Patient BC characteristics were coded based on the American Joint Committee on Cancer (AJCC) Cancer Staging Manual Eighth Edition disease specific rules for classification. Investigators were blinded to initial documentation of the explanatory variables in the EMRs.

### Statistical methods

We compared characteristics of patients between the 4 age groups using analysis of variance (ANOVA) or non-parametric Wilcoxon tests for continuous variables (based on distribution properties) and chi-square tests for categorical variables. For mortality comparisons, we constructed a dataset of expected survival probabilities for women in the US general population over a 10-year period, matched by the specific age and race distributions in our BC cohort. The expected survival probabilities were based on 2017 data from SEER (SEER 2020), and results were virtually identical when based on US general population survival probabilities reported by the US Social Security Administration [14]. Survival probabilities based on SEER data were also used as validation of a prior study [15]. To illustrate the approach, for a woman 50 years of age, the 1-year expected survival probability was 0.99690, and then at age 51, the subsequent 1-year expected survival probability was 0.99661, thereby resulting in a conditional 2-year survival probability of 0.99351 (i.e., 0.99690 × 0.99661). The corresponding 2-year risk of mortality (1 – survival probability) is 0.00649 (0.65%), and this type of calculation was used to generate expected number of deaths for a given population size and person years of observation.

For each patient, we determined the length of observation based on the time from BC diagnosis to death of any cause, or the patient’s last known date of contact. Kaplan-Meier survival curves stratified by the 4 age groups were plotted over different annual intervals. This was followed by calculation of observed to expected number of deaths at each year of age and each year of follow-up (out to 10 years) among patients in the study cohort. The age-specific observed and expected deaths were then aggregated to all patients within each study-defined age group (20 to 44, 45 to 55, 56 to 69, 70 to 90), and SMRs and 95% confidence intervals were calculated [16].

Within each of the four age groups, Cox proportional hazards regression models were fit to identify factors independently associated with 5-year mortality after cancer diagnosis. Race, marital status, and area deprivation index score (a scale of neighborhood rank by disadvantage of a given census block) [17] were included with subsequent stepwise selection of BC specific variables (at *p* < 0.01) including stage, tumor grade, number of positive LN’s, evidence of metastases, hormone receptor status, and treatment received; all at the time of diagnosis. Variables selected for the age group 20 to 44 years were fit in the remaining age group models, with tests of statistical interaction performed to assess whether BC characteristics prognostic of 5-year mortality in the age 20 to 44 group conferred differential effect (effect modification) compared to women 45 years and older at the time of diagnosis. We did not impute missing values in any analyses. Methods and results are reported in accordance with The REporting of studies Conducted using Observational Routinely-collected Health Data (RECORD) statement [18] (Supplementary Table 1).

### Ethics approval

Our study received formal ethics approval by the UPMC Ethics and Quality Improvement Review Committee (Project ID 2882), the ethics/oversight body for ensuring patient confidentiality and consent (including waiver of consent) and analysis and dissemination of deidentified data within the UPMC system.

### Availability of data

Part of the data used in this manuscript is publicly available and can be found on the US Surveillance, Epidemiology, and End Results (SEER) program of the National Cancer Institute.

## RESULTS

Among 52,509 patients, respective numbers of patients by age group were: 20 to 44 years (*n* = 5,589; 10.6%), 45 to 55 years (*n* = 12,499; 23.8%), 56 to 69 years (*n* = 19,035; 36.3%), and 70 to 90 years (*n* = 15, 386; 29.3%). Ninety-two percent or more of patients were of white race across the 4 age groups (Table 1). With increasing age, the proportion of patients with white race increased with a corresponding decrease in patients of black race. As expected, co-morbidity rates were significantly more frequent, as were rates of selected medication use with increasing age. In addition, we found that mean vitamin D levels were progressively higher across age groups (Table 1).

**Table 1:** Demographic and clinical characteristics of breast cancer patients by age at diagnosis.

**Characteristic at time of diagnosis?**	**20 to 44**	**45 to 55**	**56 to 69**	**70 to 90**	***p*-value**
	**(*N* = 5589)**	**(*N* = 12499)**	**(*N* = 19035)**	**(*N* = 15386)**	
Demographics					
Race of patient, No. (%)					
White	5097 (91.7)	11621 (93.4)	17827 (94.1)	14512 (95.0)	<.001
Black	3778 (6.8)	687 (5.5)	975 (5.1)	698 (4.6)	
Other	83 (1.5)	133 (1.1)	137 (0.7)	58 (0.4)	
Hispanic ethnicity, No. (%)	21 (0.4)	47 (0.4)	70 (0.4)	32 (0.2)	.03
Selected Comorbidities, No. (%)					
Coronary artery disease	4 (0.2)	38 (1.0)	304 (4.5)	5559 (12.0)	<.001
Chronic kidney disease	4 (0.2)	15 (0.4)	101 (1.5)	253 (5.4)	<.001
Congestive heart failure	5 (0.3)	42 (1.1)	134 (2.0)	284 (6.1)	<.001
Chronic obstructive pulmonary disease	38 (2.3)	117 (3.0)	429 (6.4)	439 (9.5)	<.001
Depression	149 (9.0)	446 (11.6)	771 (11.4)	544 (11.7)	.02
Diabetes	38 (2.3)	175 (4.5)	818 (12.1)	866 (18.6)	<.001
Gastroesophageal reflux disease	140 (8.4)	438 (11.4)	1169 (17.3)	1001 (21.6)	<.001
Hypertension	110 (6.6)	673 (17.5)	2244 (33.3)	2365 (50.9)	<.001
Obesity	412 (24.8)	1308 (33.9)	2753 (40.9)	1679 (36.2)	<.001
Selected Anthropometric and Lab Values					
Total cholesterol^*^, mean, median	165.1, 162.5	170.4, 162.5	173.5, 162.5	171.1, 162.5	<.001
Vitamin D, mean, SD	25.2, 16.8	26.6, 15.3	31.2, 17.2	33.9, 16.7	<.001
Neutrophil to lymphocyte ratio^*^, mean, median	3.0, 2.3	3.0, 2.3	2.9, 2.3	3.2, 2.4	<.001
Neutrophil to lymphocyte ratio, No. (%)					
< = 3.0	973 (72.3)	2132 (72.5)	3700 (72.4)	2182 (66.2)	<.001
3.0 to 6.0	305 (22.7)	647 (22.0)	1100 (21.5)	894 (27.1)	
>6.0	67 (5.0)	161 (5.5)	307 (6.0)	220 (6.7)	
Systemic immune-inflammation index^*^, mean, median	784, 582	821, 587	752, 568	776, 579	.005
Selected Medications, No. (%)					
Anti-depressant	357 (21.5)	1032 (26.8)	1808 (26.8)	1127 (24.3)	<.001
Anti-platelet	31 (1.9)	308 (8.0)	1524 (22.6)	1755 (37.8)	<.001
Aspirin	62 (3.7)	439 (11.4)	1914 (28.4)	2158 (46.5)	<.001
Corticosteroids	222 (13.4)	573 (14.9)	1258 (18.7)	946 (20.4)	<.001
Metformin	40 (2.4)	186 (4.8)	749 (11.1)	546 (12.4)	<.001
Opioids	179 (10.8)	496 (12.9)	1036 (15.4)	810 (17.4)	<.001
Statin	45 (2.7)	485 (12.6)	2229 (33.1)	2184 (47.0)	<.001

^*^Assessed by non-parametric Kruskal-Wallis test.

Inflammation is associated to aging mechanisms and cancer development [19]. The NLR and the SII are biomarkers used to indirectly investigate the influence of inflammation with cancer prognosis [20, 21]. In this regard, higher NLR ratios and SII index are in some studies associated with poorer survival. We therefore analyzed these parameters in our cohort and found that with increased age, more patients had NLRs >6.0. The SII was nominally higher among women <55 years of age, although a linear trend with higher SII values as women aged was not observed (Table 1).

The AJCC stage of BC was not available in the records (undetermined status) in about a quarter of all patients. Removing women with undetermined status, the respective percentages of women with Stage 0/1 disease across the 4 age groups were 54.4%, 67.1%, 71.4%, and 69.1% (*p* < 001). The youngest group (ages 20–44) had overall higher stages of BC, although elderly women (ages 70–90) had the highest prevalence of stage 4 disease at presentation. Women in the elderly age group were also more likely to have intermediate grade II BC as compared to the other age groups (Table 2). Similarly, women in the elderly group were more likely to be diagnosed with lower tumor grade and well-differentiated tumors. Youngest women were more likely to have larger tumor size and regional LN involvement upon diagnosis (*p* < 0.001). Elderly women were more likely to be diagnosed with T4 than other age groups (*p* < 001) (Table 2). As women aged, they were less likely to have regional LN involvement. The prevalence of tumor hormone receptor positivity was estrogen receptor (ER) negative (total *n* = 31,852; 16.4%), human epidermal growth factor receptor 2 (HER2) negative (total *n* = 15,662; 85.0%), progesterone receptor (PR) negative (total *n* = 31,686; 25.5%), and triple negative (total *n* = 52,509; 7.9%). Younger women (<45 years) were more likely to have ER negative and Her2 positive tumors (Table 2).

**Table 2:** Pathologic breast cancer characteristics by age at diagnosis.

**Characteristic at time of diagnosis?**	**20 to 44**	**45 to 55**	**56 to 69**	**70 to 90**	***p*-value**
	**(*N* = 5589)**	**(*N* = 12499)**	**(*N* = 19035)**	**(*N* = 15386)**	
AJCC Stage – Clinical, No. (%)					
Stage 0	822 (19.7)	2285(24.8)	2983 (21.4)	1689 (15.9)	<.001
Stage 1	1450 (34.8)	3904 (42.3)	6979 (50.0)	5631 (53.2)	
Stage 2	1378 (33.0)	2172 (23.5)	2648 (19.0)	2058 (19.4)	
Stage 3	369 (8.8)	526 (5.7)	635 (4.6)	516 (4.9)	
Stage 4	154 (3.7)	339 (3.7)	705 (5.0)	698 (6.6)	
Tumor grade, No. (%)					
Grade 1	467 (11.0)	1622 (16.9)	2795 (19.5)	2517 (21.8)	<.001
Grade 2	1702 (40.0)	4304 (45.0)	6813 (47.5)	5694 (49.4)	
Grade 3	2088 (49.0)	3642 (38.1)	4730 (33.0)	3316 (28.8)	
Tumor grade description, No. (%)					
Grade I: Well differentiated	477 (11.1)	1642 (17.0)	2807 (19.4)	2533 (21.9)	<.001
Grade II: Moderately differentiated	1721 (39.9)	4353 (45.0)	6865 (47.5)	5728 (49.4)	
Grade III: Poorly differentiated	2112 (49.0)	3678 (38.0)	4770 (33.0)	3332 (28.7)	
Primary tumor definition (clinical), No. (%)					
Carcinoma *in situ*	877 (20.9)	2344 (25.7)	3052 (22.9)	1742 (17.4)	<.001
Tumor 2 cm or less	1494 (35.6)	3933 (43.1)	6654 (49.9)	5310 (52.8)	
Tumor >2 cm but <5 cm	1306 (31.1)	2049 (22.4)	2556 (19.2)	2072 (20.6)	
Tumor >5 cm	368 (8.8)	480 (5.3)	519 (3.9)	339 (3.4)	
Pathologic grade T4	151 (3.6)	326 (3.6)	539 (4.1)	587 (5.8)	
Regional LN’s (clinical), No. (%)					
No regional LN metastases	3293 (77.4)	8064 (85.1)	12468 (87.1)	9400 (87.7)	<.001
N1	818 (19.2)	1174 (12.4)	1412 (9.9)	1024 (9.6)	
N2	95 (2.2)	156 (1.7)	276 (1.9)	200 (1.9)	
N3	51 (1.2)	80 (0.8)	153 (1.1)	91 (0.8)	
Distant metastases, No. (%)					
No evidence of distant metastases	4179 (96.5)	9375 (96.6)	13995 (95.2)	10457 (93.8)	<.001
Distant metastases detected (cM1)	141 (3.3)	316 (3.3)	642 (4.4)	658 (5.9)	
Histological metastases in distant organs (pM1)	9 (0.2)	14 (0.1)	57 (0.4)	33 (0.3)	
Number of positive nodes, No. (%)					
None	2710 (61.8)	6338 (67.6)	10557 (73.6)	7306 (75.1)	<.001
1 to 3	1123 (25.6)	2098 (22.4)	2613 (18.2)	1634 (16.8)	
4 to 10	404 (9.2)	689 (7.4)	808 (5.6)	564 (5.8)	
More than 10	146 (3.3)	249 (2.7)	361 (2.5)	227 (2.3)	
Receptor Status, No. (%)					
Positive estrogen receptor (ER)	2435 (77.7)	6122 (81.4)	10081 (84.1)	7983 (86.7)	<.001
Positive human epidermal growth factor receptor (HER2)	344 (23.3)	607 (18.2)	882 (14.3)	515 (11.0)	<.001
Positive progesterone receptor (PR)	2255 (72.3)	5569 (74.4)	8769 (73.6)	7016 (76.6)	<.001
Triple negative (ER-, PR-, HER2-)	444 (7.9)	863 (6.9)	1558 (8.2)	1002 (6.5)	<.001

Missing cases (%) for characteristics that could not be assessed or that status was undetermined: AJCC Stage – Clinical (27.7%), Tumor grade (24.4%), Tumor grade description (23.8%), Primary tumor definition (clinical) (30.1%), Regional LN’s (clinical) (26.2%), Distant metastases (24.1%), Number of positive nodes (28.0%), Positive estrogen receptor (ER) (39.3%), Positive human epidermal growth factor receptor (HER2) (70.1%), Positive progesterone receptor (PR) (39.7%). The Triple negative (ER-, PR-, HER2-) percentages are based on the full sample (including missing information) with the numerator consisting of documented negative classification for all 3 receptors.

Fifty-one percent of women 20–44 years had one or more LN’s removed which was significantly higher than all other age groups (Table 3). Younger women were also more likely to have received chemotherapy regardless of stage at diagnosis and tumor grade (Supplementary Table 2), and less likely to have received hormone therapy (*p* < 0.001). The elderly group was least likely to have received radiation treatment (*p* < 001).

**Table 3:** Treatment approaches of breast cancer patients by age at diagnosis.

**Characteristic**	**20 to 44**	**45 to 55**	**56 to 69**	**70 to 90**	***p*-value**
	**(*N* = 5589)**	**(*N* = 12499)**	**(*N* = 19035)**	**(*N* = 15386)**	
Lymph node surgery, No. (%)					
None	979 (18.1)	2826 (23.2)	4200 (22.7)	5217 (35.2)	<.001
Sentinel lymph node biopsy	1665 (30.8)	4059 (33.3)	6996 (37.9)	4372 (29.5)	
1 or more lymph nodes removed	2768 (51.1)	5314 (43.6)	7282 (39.4)	5249 (35.4)	
Staging procedure description, No. (%)					
No surgical diagnostic/staging procedure performed	1520 (27.4)	3236 (26.1)	4498 (23.9)	4427 (29.3)	<.001
Biopsy done to the primary site	3977 (71.7)	9059 (72.9)	14122 (74.9)	10490 (69.4)	
Other	49 (0.9)	127 (1.0)	235 (1.2)	205 (1.4)	
Chemotherapy received, No. (%)	3366 (61.7)	5569 (45.8)	6124 (33.2)	2024 (13.6)	<.001
Radiation modality description, No. (%)					
No radiation treatment	2491 (44.6)	4737 (37.9)	6949 (36.5)	7299 (47.4)	<.001
Brachytherapy	15 (0.3)	157 (1.3)	380 (2.0)	230 (1.5)	
External beam, NOS	999 (17.9)	2080 (16.6)	2803 (14.7)	2202 (14.3)	
IMRT	529 (9.5)	1529 (12.2)	2569 (13.5)	1558 (10.1)	
Photons	775 (13.9)	2088 (16.7)	2930 (15.4)	1927 (12.5)	
Other radiation treatment	687 (12.3)	1769 (14.2)	3161 (16.6)	1937 (12.6)	
Undetermined	93 (1.7)	139 (1.1)	243 (1.3)	233 (1.5)	
Days of radiation treatment, mean, SD	46.0, 76.2	43.5, 66.9	41.4, 86.0	39.3, 56.5	<.001
Hormone therapy received, No. (%)	2867 (55.2)	7440 (63.4)	12342 (68.0)	9496 (64.2)	<.001

Abbreviations: NOS: Not otherwise specified; IMRT: Intensity-modulated radiation therapy.

**Figure 1:**
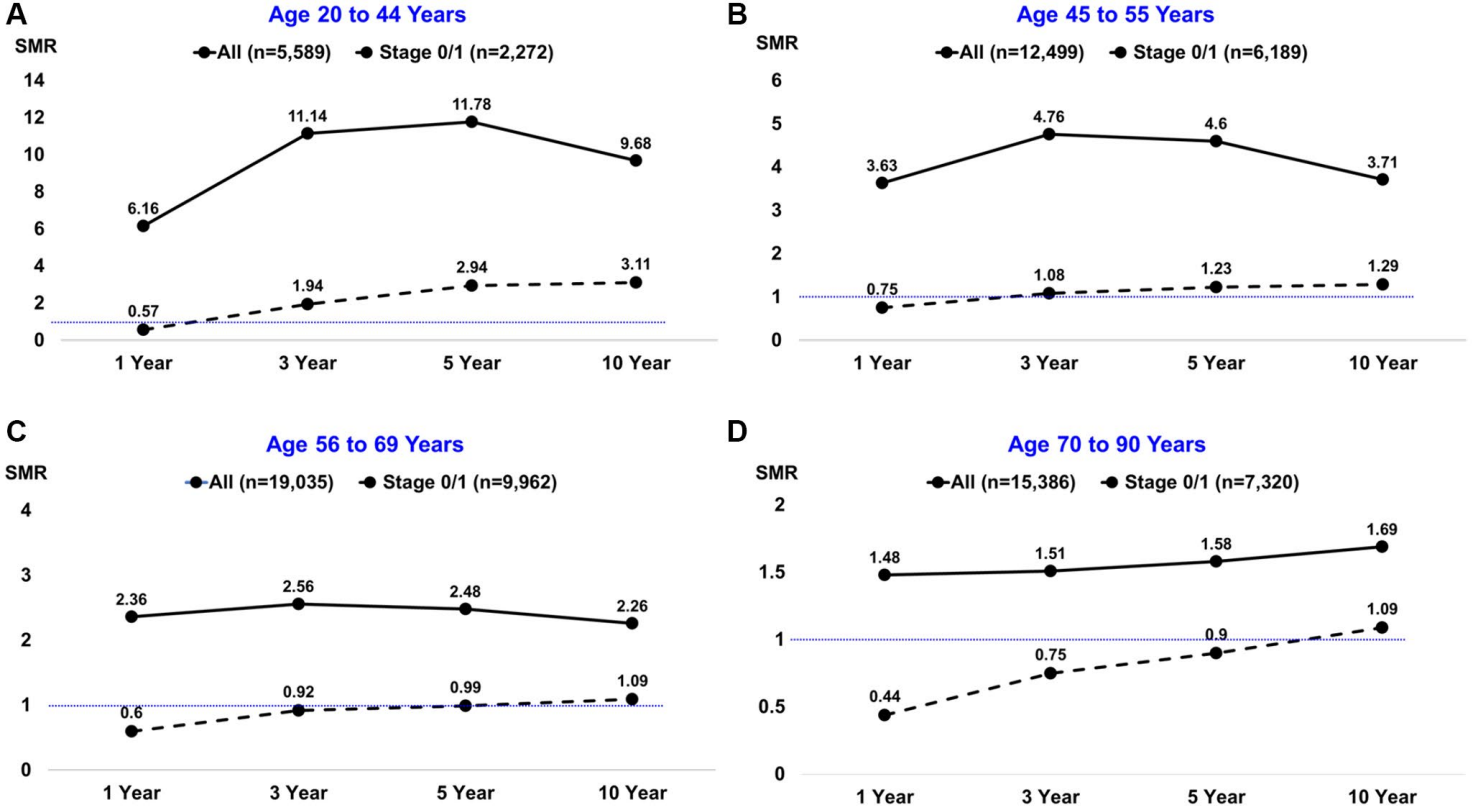
**Standardized mortality ratios (SMRs) by age group at breast cancer diagnosis and different follow-up intervals.** The solid black line depicts all patients; the dashed black line depicts the subset of patients diagnosed with stage 0/1 disease. The horizontal thin blue line depicts SMRs below or above the null value of 1.0.

SMRs of all-cause mortality after BC diagnosis were determined by age group and are reported in Supplementary Table 3, with cases limited to Stage 0/1 disease listed in Supplementary Table 4. Notably higher SMRs occurred in BC patients aged 20 to 44 years, with a peak of 11.78 at 5 years post diagnosis compared to 4.60 in the age 45 to 55 cohort, 2.48 in the age 56 to 69 cohort, and lowest at 1.58 in ages 70 to 90 (Figure 1, solid lines). Similar trends of decreasing SMRs as age increased were observed when the analysis was limited to patients diagnosed with Stage 0/1 disease (Figure 1, dashed lines). In patients diagnosed with Stage 0/1 disease, trend towards an SMR <1 was observed at 1-year follow-up for all cohorts. The SMR remained significant at <1 at 3- and 5-year follow-up for patients aged 70 and older, with 10-year mortality rates reaching an SMR similar to the general population (Figure 1A–1D, Supplementary Table 4), likely due to age-related natural death.

**Figure 2:**
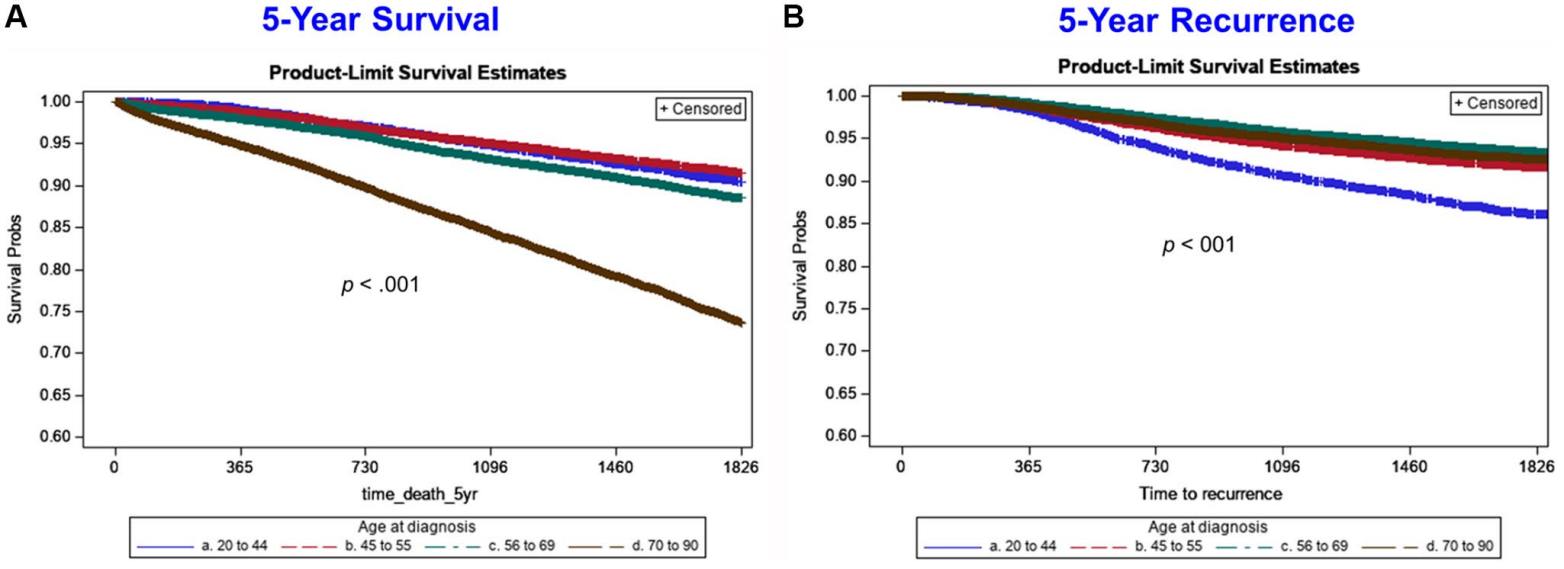
**Five-year Kaplan-Meier survival curves by age group for all-cause mortality (left side) and breast cancer recurrence (right side).** Age group lines are: blue line (20 to 44 years), red/orange line (45 to 55 years), green line (56 to 69 years), brown line (70 to 90 years).

Rates of 5-year overall survival were similar in patients <70 years of age, whereas women diagnosed at ages 70 to 90 years had worse 5-year overall survival outcomes compared to the rest of age groups. We also found that rates of 5-year BC recurrence were similar in patients aged ≥45 years, while women diagnosed at ages 20 to 44 years (Figure 2) had higher recurrence rates than the other age groups. In Cox regression models stratified by age (Table 4), factors independently associated with worse 5-year mortality among women diagnosed at age 20 to 44 years included: HER2 negative BC, tumor grades 2 and 3, stage 2 or higher disease, and regional LN metastases (all hazard ratios >2.3). The adjusted hazard ratio (HR) of 5-year mortality by age for women diagnosed at age 20 to 44 years with HER2 negative BC (HR = 2.63; 95% CI: 1.46 to 4.74) was markedly higher than for women diagnosed at age 45 to 55 years (HR = 1.67; 95% CI: 1.18 to 2.36), 56 to 69 years (HR = 1.67; 95% CI: 1.26 to 2.21), and 70 to 90 years (HR = 1.06; 95% CI: 0.86 to 1.30). Similarly, the adjusted hazard ratio (HR) for women diagnosed at age 20 to 44 years for tumor grade 3 (HR = 7.22; 95% CI: 2.29 to 22.78) was substantially higher than for women diagnosed at age 45 to 55 years (HR = 2.52; 95% CI: 1.64 to 3.85), 56 to 69 years (HR = 1.37; 95% CI: 1.12 to 1.67), and 70 to 90 years (HR = 1.37; 95% CI: 1.20 to 1.55).

**Table 4:** Adjusted^a^ hazard ratios of factors associated with 5-year mortality by age at breast cancer diagnosis.

	**(Age 20 to 44)**		**(Age 45 to 55)**		**(Age 56 to 69)**		**(Age 70 to 90)**	
	***n* = 5001, 403 events**		***n* = 11285, 791 events**		***n* = 17377, 1619 events**		***n* = 14247, 3120 events**	
Factor	HR	95% CI	HR	95% CI	HR	95% CI	HR	95% CI
Black race	1.03	(0.72 to 1.45)	1.20	(0.95 to 1.53)	1.37	(1.15 to 1.63)	1.13	(0.97 to 1.31)
Estrogen receptor negative	1.02	(0.61 to 1.71)	1.13	(0.84 to 1.52)	1.22	(0.99 to 1.51)	1.24	(1.05 to 1.46)
HER2 negative	2.63	(1.46 to 4.74)	1.67	(1.18 to 2.36)	1.67	(1.26 to 2.21)	1.06	(0.86 to 1.30)
Progesterone receptor negative	1.36	(0.83 to 2.23)	1.51	(1.14 to 2.00)	1.04	(0.86 to 1.24)	1.26	(1.11 to 1.43)
Tumor grade 2 (vs. grade 1)	3.93	(1.24 to 12.52)	1.87	(1.22 to 2.87)	0.94	(0.77 to 1.14)	1.08	(0.96 to 1.21)
Tumor grade 3 (vs. grade 1)	7.22	(2.29 to 22.78)	2.52	(1.64 to 3.85)	1.37	(1.12 to 1.67)	1.37	(1.20 to 1.55)
AJCC clinical Stage 2 (vs. 0,1)	2.30	(1.55 to 3.39)	3.36	(2.57 to 4.39)	2.52	(2.11 to 3.00)	2.39	(2.13 to 2.67)
AJCC clinical Stage ¾ (vs. 0,1)	6.31	(4.22 to 9.43)	8.14	(6.15 to 10.79)	5.96	(4.92 to 7.22)	4.61	(4.03 to 5.28)
Lymph node positive (1–3)	0.96	(0.62 to 1.50)	1.17	(0.84 to 1.62)	1.33	(1.05 to 1.69)	1.44	(1.20 to 1.72)
Lymph node positive (4 or more)	1.78	(1.13 to 2.80)	2.54	(1.80 to 3.57)	1.83	(1.42 to 2.36)	2.01	(1.65 to 2.45)
No distant disease (clinical)	0.38	(0.28 to 0.52)	0.38	(0.30 to 0.47)	0.41	(0.35 to 0.48)	0.60	(0.54 to 0.68)
Regional lymph node positive + (pathological)	2.43	(1.59 to 3.71)	1.62	(1.18 to 2.23)	1.27	(1.01 to 1.60)	1.13	(0.95 to 1.33)
Chemotherapy received	1.79	(1.26 to 2.55)	1.02	(0.83 to 1.25)	1.03	(0.91 to 1.17)	0.72	(0.64 to 0.81)
Endocrine therapy received	0.36	(0.28 to 0.47)	0.49	(0.41 to 0.59)	0.55	(0.48 to 0.61)	0.76	(0.70 to 0.83)
Immunotherapy received	0.71	(0.44 to 1.15)	0.82	(0.59 to 1.14)	0.75	(0.57 to 0.99)	0.75	(0.58 to 0.96)

Abbreviations: HER2: Human epidermal growth factor receptor; AJCC: American Joint Committee on Cancer. ^a^Cox proportional hazards regression.

Among patients who died within 10 years of diagnosis, recurrence rates are significantly higher in the cohort of patients aged 20–44 and gradually decreasing with age. While this precludes definitive conclusions regarding breast cancer specific survival, the higher rates of recurrence seen in the younger cohort implies higher mortality rates due to presence of disease as compared to patients aged 70–90 who died within 10 years that had substantially lower recurrence rates and thus mortality more likely secondary to other comorbidities and/or age-related mortality (Supplementary Figure 1).

## DISCUSSION

Incidence of BC is expected to rise by 50% by 2050 [22]. It is expected that these increased rates will be reflected primarily among post-menopausal women aged 70–84, particularly in those with ER+ disease, due to a better overall survival in the aging population [2]. An enhanced understanding of the underlying biological processes in elderly BC is critical to delineate the role of therapeutic intervention in this population with increased frailty.

In this study, we show better observed to expected mortality rates for BC patients as age increases, and an apparent more favorable 5-year all-cause mortality risk (SMR <1) in patients diagnosed with early-stage disease (AJCC Stage 0/1) after the age of 70 as compared to the general population. Prior work from twenty years ago demonstrated similar trends in observed to expected mortality rates as a function of age with a SEER-derived matched control group from the general population [15]. It is noteworthy that these mortality observations remain stable in despite advances in BC treatment over the past two decades. This may suggest that there may be an underlying biology of BC in aging women that is unchanged by therapeutic advances.

Our results show that black women are diagnosed with BC earlier in life as compared to white women. This dual distribution of BC diagnosis by age and race was in conjunction with a prior study [23], where non-white women (including Hispanic and Asian) also tended to present with more advanced disease at diagnosis than white women [23]. These differences may be due to higher genomic and biologic tumor heterogeneity including basal-like features, and/or socioeconomical disparity among the non-white population [24, 25].

We report progressively decreasing SMR with each increasing decade of age of BC diagnosis. This effect is maintained even when limiting analysis to *in situ* and early-stage disease (Stage 0/1). Surprisingly, with a stage 0/1 BC diagnosis in women between the ages of 70–90 the SMR remained lower than 1, indicating a survival advantage with BC diagnosis, heralding an association to a likely protective effect with tumor development that is in sharp contrast to the effect of diagnosis in younger women. A trend to SMR<1 noted for all ages of patients with Stage 0/1 disease at 1-year follow-up is likely attributed to closer medical monitoring and access to care in the early timeframe status post cancer diagnosis. Collectively, our data support the presence of a differential clinical entity with an inherently disparate biology observed in elderly patients with BC.

As described, SII and NLR are proposed to be prognostic in many tumor subtypes and may reflect underlying host immunity (27–29). In our study, the highest SII were in our pre-menopausal cohorts (ages 20–44 and 45–55). More importantly, elderly patients had more frequently NLRs >6. The presence of increased NLRs as women age may suggest the presence of evolving immune profiles underlying the development of BC. This may influence the disease phenotype as women age and could influence the decreased SMR <1 at five years seen in our 70–90-year age cohort with early-stage disease [26–28].

Additionally, we report that 5-year survival rates were worse in the 70–90 age group compared to younger women. Recurrence rates of women aged 20–44 were also higher compared to women ≥45 years of age; both findings were in line with prior reports [29–31]. As seen in the present study and discussed previously, underlying comorbidities and less invasive treatments may partly explain the higher mortality found in the 70–90 age group [29]. Higher rates of recurrence among younger women could be explained by the higher percentage of ER-/PR- tumors, decreased BC awareness at younger ages and the presence of familial mutations [31].

Limitations of our analysis include the retrospective data collection (i.e., data were not collected for research purposes), and missing data in some patients on cancer staging, tumor characterization, disease progression, and hormone receptor status. In addition, our results are from a single heath care system which precludes generalizability to the US population at large. We were also unable to discern cause of death, and therefore could not evaluate BC-specific mortality. Finally, the 30-year period of data collection (1990 to 2020) has included a large percentage of patients who were treated decades ago prior to more recent advances in the treatment of BC.

In summary, we report data indicative of a more indolent disease in BC patients with advanced age. Women with early breast cancer diagnosed at ages 70 and older had better overall 5-year survival rates than the general population, as demonstrated by low SMRs (<1). These findings suggest that BC in the elderly could be inherently different from the more clinically aggressive disease observed in younger patients. While the development of BC significantly worsens the observed to expected mortality of younger women, it may potentially serve as a factor linked to better survival in the elderly, especially in cases where stage 0/1 BC is diagnosed. Further studies are needed to better understand the underlying cues of these findings.

## Supplementary Materials

Supplementary Figure 1

Supplementary Table 1

Supplementary Tables
