# Supplementary material for: Breast cancer mortality as a function of age
Source: Aging (Albany NY). 2022 Feb 8;14(3):1186–99. doi: 10.18632/aging.203881 (PMC8876898; doi:10.18632/aging.203881)
Supplement: Supplementary Figure 1 [file aging-14-203881-s001.pdf]

SUPPLEMENTARY FIGURE

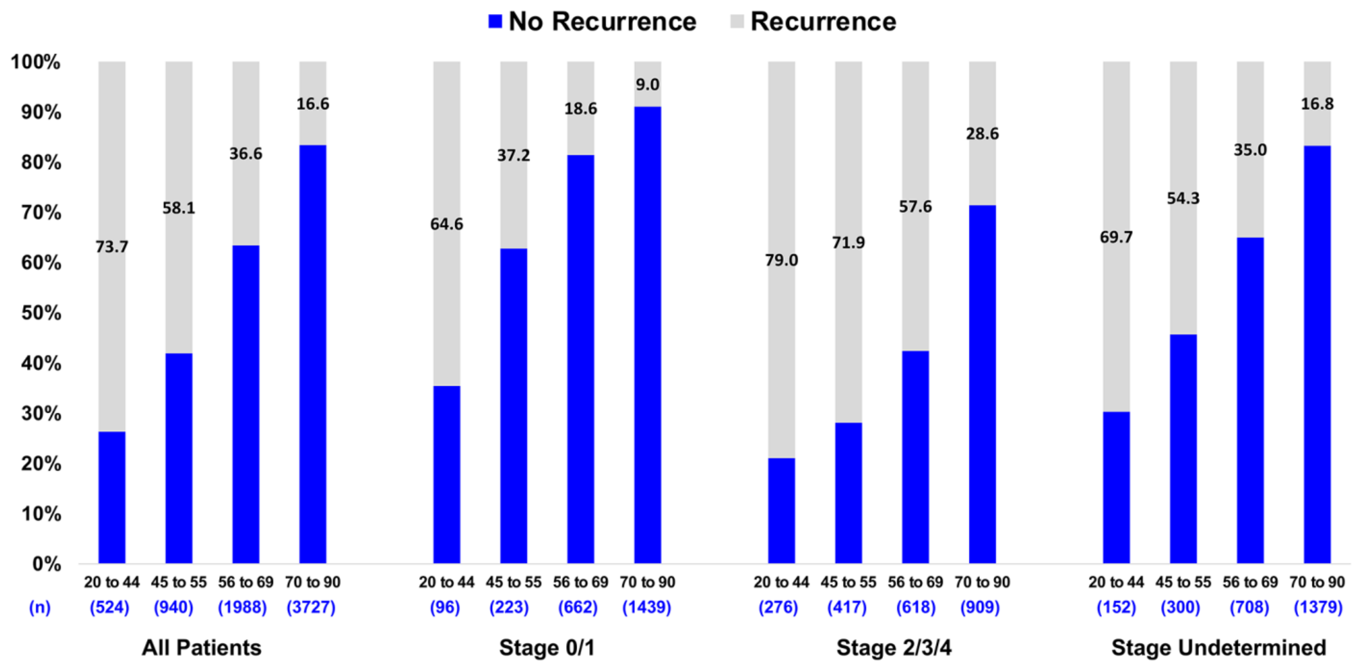

Supplementary Figure 1. 10-year breast cancer recurrence rate (%) among patients who died within 10 years.
