# Supplementary material for: Breast cancer mortality as a function of age
Source: Aging (Albany NY). 2022 Feb 8;14(3):1186–99. doi: 10.18632/aging.203881 (PMC8876898; doi:10.18632/aging.203881)
Supplement: Supplementary Tables [file aging-14-203881-s003.pdf]

SUPPLEMENTARY TABLES

Supplementary Table 2. Percentage of use of radiation, chemotherapy, and hormone therapy by age and severity of disease.

	Radiation Received				Chemo Received				Hormone TX Received			
	20–44	45–55	56–69	70–90	20–44	45–55	56–69	70–90	20–44	45–55	56–69	70–90
**AJCC Stage – Clinical**												
Stage 0	38.8	51.9	56.7	50.6	3.4	2.1	1.4	0.7	43.8	55.4	56.7	44.9
Stage 1	59.7	66.4	69.3	57.6	57.3	40.0	25.9	8.4	68.3	76.2	79.8	74.7
Stage 2	56.5	64.9	62.8	46.2	87.2	79.9	68.1	25.7	57.2	63.2	69.0	68.6
Stage 3	66.1	70.8	68.4	49.1	97.6	95.4	90.4	45.1	51.5	48.6	57.3	60.0
Stage 4	44.6	38.5	38.7	32.2	81.1	73.3	59.2	34.3	46.6	52.3	57.0	61.0
Undetermined	55.1	61.7	60.9	51.0	63.3	49.5	33.0	12.4	47.9	57.1	60.4	57.4
**Tumor grade**												
a. Grade 1	62.7	74.1	76.8	61.0	34.8	24.4	14.0	4.1	76.8	80.9	82.8	72.6
b. Grade 2	61.6	68.0	71.7	56.9	57.5	41.6	29.6	10.5	71.6	76.3	77.9	72.6
c. Grade 3	58.9	65.6	67.4	55.8	79.9	68.4	56.6	28.0	45.1	48.3	52.3	50.9
d. Other/missing	36.3	40.6	37.5	34.8	47.6	35.3	26.1	11.8	42.5	53.6	60.7	57.9
**Positive nodes**												
a. None	53.3	63.5	66.0	57.7	57.7	40.1	25.7	9.8	56.9	66.6	72.3	70.0
b. 1 to 3	62.6	64.9	64.8	54.7	89.9	83.8	67.6	31.6	65.3	71.2	75.3	75.4
c. 4 to 10	71.3	77.2	72.5	59.5	96.8	94.3	88.6	54.7	61.2	63.2	69.8	63.4
d. More than 10	64.5	69.2	70.0	62.8	95.9	93.4	90.7	55.3	42.2	55.2	70.3	70.0

Supplementary Table 3. Observed to expected mortality by age at BC diagnosis (patients with all stage disease at diagnosis included).

Age Group and Follow-up Period	*N* (PY-FU)	Observed Deaths	Expected Deaths	SMR	95% C.I.
Age 20 to 44 years	5,589				
1-year mortality	(5,400.2)	48	7.8	6.16	4.59 to 8.09
3-year mortality	(14,684.5)	252	22.6	11.14	9.83 to 12.59
5-year mortality	(22,458.8)	436	37.0	11.78	10.72 to 12.93
10-year mortality	(36,241.8)	700	72.3	9.68	8.99 to 10.42
Age 45 to 55 years	12,499				
1-year mortality	(11,911.2)	142	39.2	3.63	3.06 to 4.26
3-year mortality	(32,507.2)	547	115.0	4.76	4.37 to 5.17
5-year mortality	(49,856.5)	874	190.0	4.60	4.30 to 4.91
10-year mortality	(80,564.4)	1362	366.7	3.71	3.52 to 3.92
Age 56 to 69 years	19,035				
1-year mortality	(18,019.2)	367	155.3	2.36	2.13 to 2.61
3-year mortality	(47,481.7)	1121	437.9	2.56	2.41 to 2.71
5-year mortality	(71,447.5)	1742	720.0	2.48	2.37 to 2.60
10-year mortality	(111,298.2)	2925	1294.7	2.26	2.18 to 2.34

Age 70 to 90 years	15,386				
1-year mortality	(14,200.7)	771	519.5	1.48	1.38 to 1.59
3-year mortality	(36,627.7)	2105	1392.7	1.51	1.45 to 1.58
5-year mortality	(54,052.9)	3334	2113.6	1.58	1.52 to 1.63
10-year mortality	(80,727.1)	5597	3310.9	1.69	1.65 to 1.74

Abbreviation: PY-FU: Person years of follow-up censoring at time of death or date of last contact.

Supplementary Table 4. Observed to expected mortality by age at BC diagnosis (limited to patients with Stage 0/1 disease).

Age Group and Follow-up Period	*N* (PY-FU)	Observed Deaths	Expected Deaths	SMR	95% C.I.
Age 20 to 44 years	2,272				
1-year mortality	(2,208.9)	2	3.5	0.57	0.10 to 1.89
3-year mortality	(6,130.8)	19	9.8	1.94	1.20 to 2.97
5-year mortality	(9,499.2)	48	16.3	2.94	2.20 to 3.87
10-year mortality	(15,362.0)	101	32.5	3.11	2.54 to 3.76
Age 45 to 55 years	6,189				
1-year mortality	(5,983.2)	16	21.2	0.75	0.45 to 1.19
3-year mortality	(16,435.1)	64	59.0	1.08	0.84 to 1.38
5-year mortality	(25,323.4)	122	98.7	1.23	1.03 to 1.47
10-year mortality	(40,383.6)	248	191.7	1.29	1.14 to 1.46
Age 56 to 69 years	9,962				
1-year mortality	(9,578.0)	54	89.9	0.60	0.46 to 0.78
3-year mortality	(25,336.1)	216	234.4	0.92	0.80 to 1.05
5-year mortality	(38,122.5)	374	378.4	0.99	0.89 to 1.09
10-year mortality	(57,855.8)	763	696.0	1.09	1.02 to 1.18
Age 70 to 90 years	7,320				
1-year mortality	(6,914.3)	119	269.7	0.44	0.37 to 0.53
3-year mortality	(18,069.1)	493	656.4	0.75	0.69 to 0.82
5-year mortality	(26,722.0)	915	1015.6	0.90	0.84 to 0.96
10-year mortality	(38,949.8)	1764	1611.1	1.09	1.05 to 1.15

Abbreviation: PY-FU: Person years of follow-up censoring at time of death or date of last contact.
